# Pneumococcal nasopharyngeal carriage in children under 5 years of age at an outpatient healthcare facility in Novi Sad, Serbia during the COVID-19 pandemic

**DOI:** 10.1016/j.ijregi.2022.07.001

**Published:** 2022-07-07

**Authors:** Vladimir Petrović, Biljana Milosavljević, Milan Djilas, Miloš Marković, Vladimir Vuković, Ilija Andrijević, Mioljub Ristić

**Affiliations:** a Department of Epidemiology, Faculty of Medicine, University of Novi Sad, Novi Sad, Serbia; b Institute of Public Health of Vojvodina, Novi Sad, Serbia; c Department of Immunology, Faculty of Medicine, University of Belgrade, Institute of Microbiology and Immunology, Belgrade, Serbia; d Institute for Pulmonary Diseases of Vojvodina, Sremska Kamenica, Serbia

**Keywords:** Streptococcus pneumoniae, Pneumococcal serotypes, Nasopharyngeal colonization, COVID-19

## Abstract

**Objectives:**

To assess whether pneumococcal nasopharyngeal carriage among children aged 24–60 months reduced during the coronavirus disease 2019 (COVID-19) pandemic in Novi Sad, Serbia, and to investigate the overall prevalence of carriage, serotype distribution and dominant serotypes 2–3 years after the introduction of pneumococcal conjugate vaccine 10.

**Design and methods:**

This prospective, observational study was conducted in February–March 2020, September–November 2020 and April–June 2021, enabling the comparison of results in the pre-pandemic/early pandemic period with two periods during the COVID-19 pandemic. Pneumococci were identified by standard microbiological methods. Serotype identification was performed using conventional multiplex polymerase chain reaction assays.

**Results:**

Among 1623 children tested, 515 (31.7%, 95% confidence interval 29.4–34.0%) carried pneumococci. A significant increase in prevalence was found between February–March 2020 and September–November 2020 (*P*=0.0085), with no difference found between September–November 2020 and April–June 2021 (*P*=0.0524). Pneumococcal colonization was significantly higher in children who were fully vaccinated and among children who attended day care centres. The dominant serotypes were 15B, 6B, 19F, 11A, 6C, 6A, 3, 23F and 19A, representing 66.4% of all isolates.

**Conclusions:**

This study found that pneumococcal nasopharyngeal carriage in children aged 24–60 months was high before the COVID-19 pandemic, and then increased during the pandemic. This rules out a major role of COVID-19 in the suppression of carriage and, probably, transmission.

## Highlights

• The prevalence of nasopharyngeal pneumococcal carriage in children aged 24–60 months was 31.7%.
• The prevalence was high and increased during the coronavirus disease 2019 (COVID-19) pandemic.
• This ruled out a major role of COVID-19 in suppressing carriage and, probably, transmission.
• The dominant serotypes were 15B, 6B, 19F, 11A, 6C, 6A, 3, 23F and 19A.

## Introduction

Asymptomatic nasopharyngeal carriage plays an essential role in the transmission of *Streptococcus pneumoniae*, and is a prerequisite for disease development, including invasive pneumococcal disease (IPD) (Bogaert et al., 2004; Simell et al., 2012; World Health Organization, 2012). Both pneumococci and respiratory viruses, including severe acute respiratory syndrome coronavirus-2 (SARS-CoV-2), can be transmitted from person to person, and also indirectly via contact with contaminated surfaces or objects (Marks et al., 2014; Weiser et al., 2018; Leung, 2021; Marzoli et al., 2021; Morimura et al., 2021). Close contact between individuals, especially in crowded places, adds to the spread of pneumococci. Infants and young children are the main reservoir of infection, particularly when attending day care centres (World Health Organization, 2012; Numminen et al., 2015; Andrejko et al., 2021). In day care settings, pneumococci survive for hours and could be cultured from environmental surfaces, including toys (Marks et al., 2014). During the coronavirus disease 2019 (COVID-19) pandemic, there was a large reduction in pneumococcal disease in 2020, and non-pharmaceutical interventions were suggested to be responsible for reduced pneumococcal carriage, circulation and transmission, resulting in reduced pneumococcal disease (Katzow et al., 2020; Angoulvant et al., 2021; Cohen et al., 2021; Janapatla et al., 2021; Williams et al., 2021; Perniciaro et al., 2022).

Assessment of the prevalence of nasopharyngeal carriage of *S. pneumoniae* before and after introduction of the pneumococcal conjugate vaccines (PCVs) in some countries can be used to evaluate the impact of the vaccines on the epidemiology of this pathogen (World Health Organization, 2012; Adegbola et al., 2014). Until 2018, PCVs were mainly used for immunization of high-risk groups in Serbia (Petrović et al., 2016; Institute of Public Health of Vojvodina, 2019). In April 2018, mandatory routine childhood immunization with PCV10 was introduced. Primary vaccination included three doses for all children aged 2–6 months, followed by a booster dose in the second year of life (Ministry of Health of the Republic of Serbia, 2018a, Ministry of Health of the Republic of Serbia, 2018b). Pneumococcal vaccination coverage (three doses) among infants was 45% in Novi Sad in 2018, and reached 85–95% in 2019–2021. A fourth dose was given to 71–80% of children (Institute of Public Health of Vojvodina, 2019, 2020, 2021).

The primary aim of this study was to assess nasopharyngeal carriage of *S. pneumoniae* among children aged 24–60 months during the COVID-19 pandemic in Novi Sad, and to evaluate the overall prevalence of nasopharyngeal carriage, serotype distribution and dominant serotypes 2–3 years after the introduction of PCV10.

## Methods

### Study site and participants

Novi Sad is the capital and the administrative centre of Vojvodina. It has 341,624 inhabitants, 12,812 of whom are children aged 24–60 months according to the 2011 Census. This research was carried out by the Medical Faculty of Novi Sad in collaboration with the Institute of Public Health of Vojvodina and the Paediatric Department of the Primary Healthcare Centre (PHCC) in Novi Sad.

### COVID-19 and control measures in the Republic of Serbia

The first case of COVID-19 in the Republic of Serbia (henceforth 'Serbia') was recorded on 6 March 2020. On 15 March 2020, a state of emergency was declared at the national level. Strict measures were implemented, such as closing borders; movement restrictions; school, day care centre and faculty closures; and the recommendation that all citizens aged ≥65 years should stay indoors. Relaxation of measures on 22 May 2020 resulted, as expected, in an increase in the number of cases of COVID-19, leading to a second wave (Lazić et al., 2020; Pustahija et al., 2021). Day care centres were opened after the relaxation of measures, and non-pharmaceutical interventions, other than handwashing and disinfection, were not followed strictly, including wearing masks.

**Figure 1:**
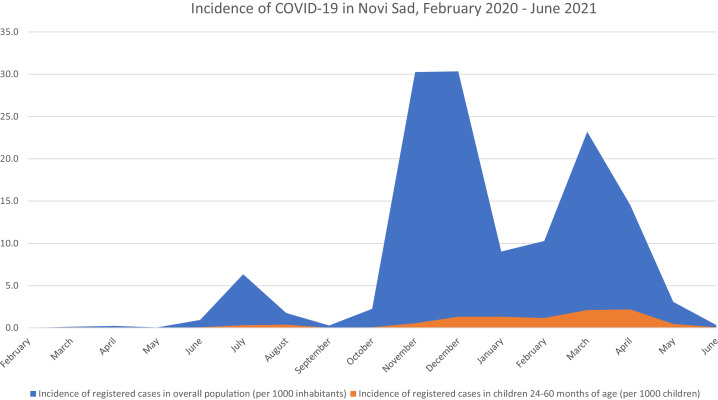
Incidence of coronavirus disease 2019 in Novi Sad, February 2020–June 2021.

Three additional epidemic waves were registered up to July 2021 in Serbia, including in the city of Novi Sad (Figure 1). In total, 45,447 cases were registered in Novi Sad between 11 March 2020 (first case) and the end of June 2021, with an overall prevalence rate of 12.6%. Among children aged 24–60 months, 130 cases were registered, with an overall prevalence rate of 1.0%.

### Study design

This prospective, population-based study was conducted among children aged 24–60 months who visited their paediatricians at the PHCC of Novi Sad (outpatient care facility). Initially, recruitment had been scheduled for the period from 15 November 2019 to 31 December 2020, but due to logistical problems during the COVID-19 pandemic, it was mainly conducted in February–March 2020, September–November 2020 and April–June in 2021, enabling the comparison of results in the pre-pandemic/early pandemic period with two periods during the pandemic.

Participants included in this study were selected and sampled (one nasopharyngeal swab per study subject) by the 10 paediatricians at the PHCC of Novi Sad. Only children who visited a physician were recruited, regardless of whether or not they had any signs of upper respiratory tract infection. However, participants were excluded if they had used antibiotics in the preceding 7 days or had a chronic and/or severe pathology. At admission, physicians interviewed parent(s)/legal guardian(s) of all subjects through face-to-face structured interviews. After providing a verbal and written explanation of the research aims, informed consent was obtained from parent(s)/legal guardian(s) before enrolment, and prior to the performance of any study-related procedures. Participants’ personal and confidential information was removed, except for demographic information including date of sampling, age, gender, number of doses and dates of administration of PCV10, and attendance at a day care centre.

### Sample collection and processing

Nasopharyngeal swabs were collected and transported within 12 h to the Institute of Public Health of Vojvodina in transport medium (Copan Venturi Transystem, Brescia, Italy). Nasopharyngeal swabs were inoculated in STGG medium, 200 μL of which was transferred to 5.0 mL Todd Hewitt broth containing 0.5% yeast extract and 1 mL of rabbit serum, and incubated at 35–37°C for 6 h. Cultured broth was plated on sheep blood agar and incubated in 5% CO_2_ at 35–37°C. After 18–24 h of incubation, plates were examined for the appearance of alpha-haemolytic colonies that were subcultured, and pneumococci were identified by optochin susceptibility and bile solubility test.

DNA extracts for polymerase chain reaction (PCR) were obtained using previously published protocols (Centers for Disease Control and Prevention, 2009; Lopardo et al., 2012). Identification of *S. pneumoniae* was achieved by PCR through amplification of the *lytA* gene using primers described by Nagai et al. (2001) and Gholamhosseini-Moghaddam et al. (2015). Positive samples were further analysed for serotype identification.

Conventional PCR assays were performed as a series of eight multiplex reactions, using schemes recommended by the Centers for Disease Control and Prevention, and primers for pneumococcal serotype deduction (Centers for Disease Control and Prevention, 2009; Carvalho et al., 2010). Further differentiation between serotypes 6A, 6B and 6C was performed using procedures and primers described by Jin et al. (2009). Confirmation of the results of multiplex PCR was done using standard strains: ATCC6305, ATCC49619, ATCC6303 and ATCC700677. The PCR products were analysed on 2% agarose gel, stained with ethidium bromide. Gel images were recorded using the BioDocAnalyze system (Analytik Jena, Jena, Germany) (Pai et al., 2006; Lopardo et al., 2012; Centers for Disease Control and Prevention, 2014). If isolates tested positive with optochin and *lytA* PCR, but tested negative for *cpsA* gene (acting as an internal control) in all serotyping PCR reactions, they were considered as non-typeable serotypes.

### Statistical analysis

Statistical analysis was performed using SPSS Version 22 (IBM Corp., Armonk, NY, USA) and MedCalc for Windows Version 12.3.0 (MedCalcSoftware, Mariakerke, Belgium). Categorical variables were compared using Chi-squared test, Chi-squared for goodness of fit, or test of proportions, as appropriate. Statistical significance was set at *P*<0.05.

## Results

### Prevalence and distribution of pneumococcal nasopharyngeal carriage among children aged 24–60 months during the COVID-19 pandemic

**Figure 2:**
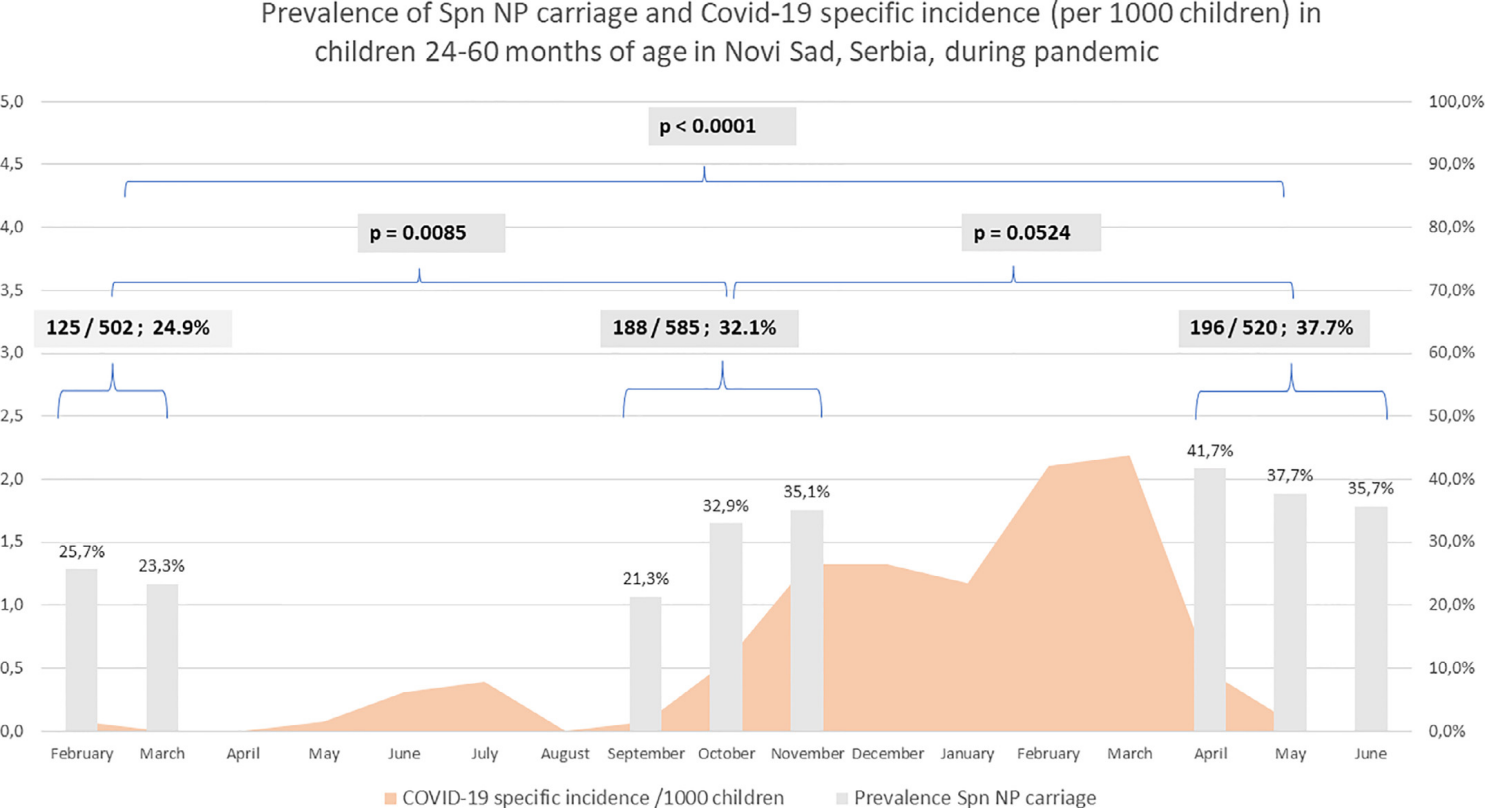
Prevalence of *Streptococcus pneumoniae* (Spn) nasopharyngeal (NP) carriage and coronavirus disease 2019 (COVID-19)-specific incidence (per 1000 children) in children aged 24–60 months in Novi Sad, Serbia during the COVID-19 pandemic. <do not include title within figure. Change ‘p =’ and ‘P <’ to ‘*P*=’ and ‘*P*<’. Use decimal points instead of commas>

Nasopharyngeal swabs were collected from 1623 children aged 24–60 months, which represented 12.7% of the population of children of the target age in Novi Sad. In total, 515 children [31.7%, 95% confidence interval (CI) 29.4–34.0%] carried *S. pneumoniae* serotypes. Prevalence of nasopharyngeal carriage was significantly higher in April–June 2021 compared with February–March 2020 (*P*<0.0001). Additionally, a significant increase in prevalence was recorded between February–March 2020 and September–November 2020 (*P*=0.0085), but no significant difference was noted between September–November 2020 and April–June 2021 (*P*=0.0524) (Figure 2). Only two of 1623 children included in the study had registered COVID-19 before the sampling period. Both were sampled in June 2021: the first child had COVID-19 in November 2020 and was negative for *S. pneumoniae*, while the second child had COVID-19 in December 2020 and harboured serotype 23A 6 months later. Prevalence was significantly lower between the observed periods in children aged 24–35 months, as well as among those aged 36–60 months (*P*=0.0007 and *P*=0.0031, respectively). *S. pneumoniae* colonization was significantly higher in children who were fully vaccinated in both age groups (*P*=0.0049 and *P*=0.0434, respectively). In addition, the majority (60.1%, 122/203) of colonized children aged 24–35 months had received four doses of PCV10, whereas the majority (85.6%, 267/312) of children aged 36–60 months were unvaccinated. Significantly higher prevalence of *S. pneumoniae* colonization was registered in both age groups and overall among children who attended day care centres. There were no differences in the prevalence of registered cases in terms of gender (Table 1).

**Table 1:** Prevalence and distribution of nasopharyngeal pneumococcal carriage among children aged 24–60 months during the coronavirus disease 2019 pandemic

Characteristics	Overall population aged 24–60 months				Children aged 24–35 months				Children aged 36–60 months			
	No. (%)	No. of positive isolates (%)	Prevalence (95% CI)	*P-*value^a^	No. (%)	No. of positive isolates (%)	Prevalence (95% CI)	*P-*value^a^	No. (%)	No. of positive isolates (%)	Prevalence (95% CI)	*P-*value^a^
Total	1623 (100)	515 (100)	31.7 (29.4–34.0)	N/A	574 (100)	203 (100)	35.4 (31.5–39.5)	N/A	1049 (100)	312 (100)	29.7 (27.0–32.6)	N/A
February–March 2020	502 (30.9)	125 (24.3)	24.9 (21.2–29.0)	**<0.0001**	161 (28.0)	36 (17.7)	22.4 (16.2–24.6)	**0.0007**	341 (32.5)	89 (28.5)	26.1 (21.5–31.1)	**0.0031**
September–November 2020	585 (36.1)	188 (36.5)	32.1 (28.4–36.1)		193 (33.6)	82 (42.4)	42.5 (35.4–49.8)		392 (37.4)	106 (34.0)	27.0 (22.7–31.7)	
April–June 2021	520 (32.0)	196 (38.1)	37.7 (33.5–42.0)		205 (35.7)	80 (39.4)	39.0 (32.3–46.1)		315 (30.0)	116 (37.1)	36.8 (31.4–42.4)	
Gender												
Female	776 (47.8)	257 (49.9)	33.1 (29.8–36.5)	0.2504	284 (49.5)	101 (49.8)	35.6 (30.0–41.5)	0.9230	492 (46.9)	156 (50.0)	31.7 (27.6–36.0)	0.1902
Male	847 (52.2)	258 (50.1)	30.5 (27.4–33.7)		290 (50.5)	102 (50.2)	35.2 (29.7–41.0)		557 (53.1)	156 (50.0)	28.0 (24.3–31.9)	
Number of doses												
0	1071 (66.0)	309 (60.0)	28.9 (26.1–31.6)	**<0.0001**	154 (26.8)	42 (20.6)	27.3 (20.4–35.1)	**0.0049**	917 (87.4)	267 (85.6)	29.1 (26.2–32.2)	**0.0434**
1–3	186 (11.5)	57 (11.1)	30.6 (24.1–37.8)		120 (20.9)	39 (19.3)	32.5 (24.2–41.7)		66 (6.3)	18 (5.7)	27.3 (17.1–39.7)	
4	366 (22.5)	149 (28.9)	40.7 (35.6–45.9)		300 (52.3)	122 (60.1)	40.7 (35.1–46.5)		66 (6.3)	27 (8.7)	40.9 (29.0–53.7)	
Attendance of day care												
No	250 (15.4)	52 (10.1)	21.2 (15.9–26.4)	**<0.0001**	130 (22.6)	26 (12.8)	20.0 (13.5–27.8)	**<0.0001**	120 (11.4)	26 (7.8)	21.7 (14.7–30.1)	**0.0394**
Yes	1373 (84.6)	463 (89.9)	33.7 (31.2–36.3)		444 (77.4)	177 (87.2)	39.9 (35.3–44.6)		929 (88.6)	286 (91.2)	30.8 (27.9–33.0)	

Significant differences (*P*<0.05) are marked in bold.

a *P*-values are based on the test of proportions.

### Streptococcus pneumoniae serotype distribution

In total, 60 (11.7%) isolates were non-typeable. Among the remaining 455 isolates, 31 *S. pneumoniae* serotypes were identified. The coverage rates of isolates by PCV were 26.4%, 27.0% and 40.8% for PCV7, PCV10 and PCV13, respectively, while serotypes present in PPSV23 alone and non-vaccine serotypes were detected in 26.6% and 21.0% of cases, respectively. Among all *S. pneumoniae* isolates, the most prevalent were 15B, 6B, 19F, 11A, 6C, 6A, 3, 23F and 19A*,* which accounted for 66.4% of the total number (342/515). Although the distributions of serotypes with regard to the different PCVs in the two age groups were similar, proportions of the isolates were significantly lower among children aged 24–35 months compared with children aged 36–60 months for all PCVs (PCV7 20.2% vs 30.4%, PCV10 20.2% vs 31.4%, PCV13 34.5% vs 44.9%), while for PPSV23, proportions were lower but the difference was not significant (56.7% vs 64.7%). Only serotype 14 had significantly higher prevalence among children aged 36–60 months compared with children aged 24–35 months (*P*=0.0385). In contrast, the proportion of non-vaccine type isolates was higher in children aged 24–35 months compared with children aged 36–60 months (24.1% vs 18.9%), but the difference was not significant. Distributions of serotypes were similar for PCV7, PCV10 and PCV13 serotypes according to attendance of a day care centre (PCV7 26.1% vs 28.8%, PCV10 26.8% vs 28.8%, PCV13 40.8% vs 36.5%), and differences were not significant. On the other hand, a significantly higher prevalence of colonization was registered for non-vaccine serotypes 6C, 15A and 23B in children who attended day care centres (Table 2).

**Table 2:** Distribution of *Streptococcus pneumoniae* serotype carriage according to age groups of participants and attendance of day care centres

Serotypes	All participants (*n*=515)	(%)	Age group					Attendance of day care centres				
			24–35 months (*n*=203)	(%)	36–60 months (*n*=312)	(%)	*P-*value^a^	Yes (*n*=463)	(%)	No (*n*=52)	(%)	*P-*value^a^
4	0	0.0	0	0.0	0	0.0	N/A	0	0.0	0	0.0	N/A
6B	48	9.3	18	8.9	30	9.6	0.7897	42	9.1	6	11.5	0.5732
9V	3	0.6	0	0.0	3	1.0	0.1534	2	0.4	1	1.9	0.1665
14	11	2.1	1	0.5	10	3.2	**0.0385**	9	1.9	2	3.8	0.3645
18C	5	1.0	1	0.5	4	1.3	0.3694	5	1.1	0	0.0	0.4477
19F	48	9.3	15	7.4	33	10.6	0.2231	44	9.5	4	7.7	0.6723
23F	21	4.1	6	3.0	15	4.8	0.3140	19	4.1	2	3.8	0.9174
Total PCV7 serotypes	136	26.4	41	20.2	95	30.4	**0.0103**	121	26.1	15	28.8	0.6756
1	0	0.0	0	0.0	0	0.0	N/A	0	0.0	0	0.0	N/A
5	1	0.2	0	0.0	1	0.3	0.4352	1	0.2	0	0.0	0.7471
7F	2	0.4	0	0.0	2	0.6	0.2693	2	0.4	0	0.0	0.6481
Subtotal	3	0.6	0	0.0	3	1.0	0.1534	3	0.6	0	0.0	0.5758
Total PCV10 serotypes	139	27.0	41	20.2	98	31.4	**0.0052**	124	26.8	15	28.8	0.7583
3	23	4.5	8	3.9	15	4.8	0.6285	19	4.1	4	7.7	0.2337
6A	30	5.8	14	6.9	16	5.1	0.3939	29	6.3	1	1.9	0.2005
19A	18	3.5	7	3.4	11	3.5	0.9517	17	3.7	1	1.9	0.5045
Subtotal	71	13.8	29	14.3	42	13.5	0.7973	65	14.0	6	11.5	0.6200
Total PCV13 serotypes (PCV vaccine serotypes)	210	40.8	70	34.5	140	44.9	**0.0191**	189	40.8	19	36.5	0.5494
10A	10	1.9	4	2.0	6	1 .9	0.9360	9	1.9	1	1.9	1.0000
11A	42	8.2	15	7.4	27	8 .7	0.5994	37	8.0	5	9.6	0.6897
12F	1	0.2	1	0.5	0	0 .0	0.2117	1	0.2	0	0.0	0.7471
15B	76	14.8	37	18.2	39	12.5	0.0749	72	15.6	4	7.7	0.1286
17F	1	0.2	1	0.5	0	0.0	0.2117	1	0.2	0	0.0	0.7471
22F	3	0.6	1	0.5	2	0.6	0.8820	3	0.6	0	0.0	0.5758
33F	3	0.6	0	0.0	3	1.0	0.1534	3	0.6	0	0.0	0.5758
9N	1	0.2	0	0.0	1	0.3	0.4352	1	0.2	0	0.0	0.7471
Subtotal PPSV23 serotypes	137	26.6	59	29.1	78	25.0	0.3040	127	27.4	10	19.2	0.2048
Total PPSV23 serotypes^b^	317	61.6	115	56.7	202	64.7	0.0685	287	61.9	30	57.7	0.5555
6C	36	7.0	18	8.9	18	5.8	0.1789	36	7.8	0	0.0	**0.0369**
7C	13	2.5	5	2.5	8	2.6	0.9441	11	2.4	2	3.8	0.5434
15A	12	2.3	4	2.0	8	2.6	0.6617	8	1.7	4	7.7	**0.0063**
23B	11	2.1	5	2.5	6	1.9	0.6457	7	1.5	4	7.7	**0.0033**
21	8	1.6	4	2.0	4	1.3	0.5335	6	1.3	2	3.8	0.1672
23A	8	1.6	4	2.0	4	1.3	0.5335	8	1.7	0	0.0	0.3439
35B	8	1.6	4	2.0	4	1.3	0.5335	7	1.5	1	1.9	0.8244
24F	5	1.0	3	1.5	2	0.6	0.3052	5	1.1	0	0.0	0.4477
10F	3	0.6	0	0.0	3	1.0	0.1534	3	0.6	0	0.0	0.5758
35F	2	0.4	1	0.5	1	0.3	0.7183	2	0.4	0	0.0	0.6481
34	1	0.2	1	0.5	0	0.0	0.2117	1	0.2	0	0.0	0.7471
39	1	0.2	0	0.0	1	0.3	0.4352	1	0.2	0	0.0	0.7471
Non-vaccine serotypes	108	21.0	49	24.1	59	18.9	0.1569	95	20.5	13	25.0	0.4501
Non-typeable isolates	60	11.7	25	12.3	35	11.2	0.7039	52	11.2	8	15.4	0.3707

Significant differences (*P*<0.05) are marked in bold.

a *P*-values are based on the test of proportion.

b Without 6A serotype.

According to the sampling period, there were no significant differences in the vast majority of serotypes, except for non-vaccine serotype 7C which was far more prevalent in April–June 2021 (5.1%) compared with February–March 2020 (0.8%) and September–November 2020 (1.1%) (Table 3).

**Table 3:** Distribution of *Streptococcus pneumoniae* serotype carriage according to sampling period

Serotypes	February–March 2020 (*n*=125)		September–November 2020 (*n*=188)		April–June 2020 (*n*=196)		*P-*value^a^
	Positive	(%)	Positive	(%)	Positive	(%)	
4	0	0.0	0	0	0	0.0	N/A
6B	11	8.8	17	9	20	10.2	0.8918
9V	2	1.6	0	0	1	0.5	N/A
14	4	3.2	5	2.7	2	1.0	0.3561
18C	2	1.6	1	0.5	2	1.0	0.6424
19F	12	9.6	18	9.6	18	9.2	0.9886
23F	3	2.4	9	4.8	8	4.1	0.5619
Total PCV7 serotypes	34	27.2	50	26.6	51	26.0	0.9729
1	0	0.0	0	0	0	0.0	N/A
5	0	0.0	0	0	1	0.5	N/A
7F	0	0.0	1	0.5	1	0.5	N/A
Subtotal	0	0.0	1	0.5	2	1.0	N/A
Total PCV10 serotypes	34	27.2	51	27.1	53	27.0	0.9995
3	9	7.2	6	3.2	8	4.1	0.2303
6A	10	8.0	13	6.9	7	3.6	0.1960
19A	3	2.4	3	1.6	11	5.6	0.0725
Subtotal	22	17.6	22	11.7	26	13.3	0.3221
Total PCV13 serotypes (PCV serotypes)	56	44.8	73	38.8	79	40.3	0.5630
10A	2	1.6	3	1.6	5	2.6	0.7524
11A	9	7.2	13	6.9	19	9.7	0.5588
12F	0	0.0	1	0.5	0	0.0	N/A
15B	19	15.2	30	16.0	24	12.2	0.5554
17F	0	0.0	1	0.5	0	0.0	N/A
22F	1	0.8	1	0.5	1	0.5	N/A
33F	1	0.8	2	1.1	0	0.0	N/A
9N	0	0.0	0	0	1	0.5	N/A
Subtotal PPSV23 serotypes	32	25.6	51	27.1	50	25.5	0.9259
Total PPSV23 serotypes^b^	78	62.4	111	59.0	122	62.2	0.7664
6C	4	3.2	14	7.4	18	9.2	0.1212
7C	1	0.8	2	1.1	10	5.1	**0.0155**
15A	6	4.8	3	1.6	3	1.5	0.1167
23B	2	1.6	2	1.1	7	3.6	0.2122
21	4	3.2	2	1.1	2	1.0	0.2416
23A	3	2.4	2	1.1	3	1.5	0.6473
35B	2	1.6	4	2.1	2	1.0	0.6835
24F	2	1.6	1	0.5	2	1.0	0.6424
10F	0	0.0	1	0.5	2	1.0	N/A
35F	1	0.8	1	0.5	0	0.0	N/A
34	0	0.0	1	0.5	0	0.0	N/A
39	1	0.8	0	0	0	0.0	N/A
Non-vaccine serotypes	26	20.8	33	17.6	49	25.0	0.2018
Non-typeable isolates	11	8.8	31	16.5	18	9.2	**0.0418**

Significant differences (*P*<0.05) are marked in bold.

a Chi-squared test.

b Without 6A serotype.

### Distribution of leading Streptococcus pneumoniae serotypes according to number of doses of PCV10

Distribution of isolates according to number of doses was significantly in favour of isolates being detected among unvaccinated children, regardless of age, for all leading serotypes (≥3% prevalence), with the exception of serotype 6C. Significantly more isolates were found among unvaccinated children aged 36–60 months compared with younger children for serotypes 6B, 19F, 11A, 6C, 23F, 3 and 19A. Also, significantly more isolates were found among children aged 24–35 months than children aged 36–60 months who had received three doses of PCV10 for serotypes 15B, 6B, 19F and 11A, and among those who had received four doses for all leading serotypes (Table 4).

**Table 4:** Distribution of leading *Streptococcus pneumoniae* serotype carriage according to age group and vaccination status

Serotype	Number of doses of PCV10	All children aged 24–60 months (*n*=515)		*P-*value^a^	Age group					*P-*value^b^
					24–35 months (*n*=203)			36–60 months (*n*=312)		
		No.	% positive according to number of doses		No.	(%)	No.		(%)	
15B (*n*=76)	0	46	60.5	**<0.001**	13	6.4	33		10.6	0.1030
	1	2	2.6		0	0.0	2		0.6	0.2693
	2	0	0.0		0	0.0	0		0.0	N/A
	3	4	5.3		4	2.0	0		0.0	**0.0122**
	4	24	31.6		20	9.9	4		1.3	**<0.0001**
6B (*n*=48)	0	34	70.8	**<0.001**	5	2.5	29		9.3	**0.0024**
	1	0	0.0		0	0.0	0		0.0	N/A
	2	2	4.2		2	1.0	0		0.0	0.0770
	3	3	6.3		3	1.5	0		0.0	**0.0302**
	4	9	18.8		8	3.9	1		0.3	**0.0022**
19F (*n*=48)	0	35	72.9	**<0.001**	5	2.5	30		9.6	**0.0018**
	1	3	6.3		1	0.5	2		0.6	0.8820
	2	0	0.0		0	0.0	0		0.0	N/A
	3	4	8.3		4	2.0	0		0.0	**0.0122**
	4	6	12.5		5	2.5	1		0.3	**0.0232**
11A (*n*=42)	0	24	57.1	**<0.001**	2	1.0	22		7.1	**0.0014**
	1	1	2.4		0	0.0	1		0.3	0.4352
	2	0	0.0		0	0.0	0		0.0	N/A
	3	4	9.5		4	2.0	0		0.0	**0.0122**
	4	13	31.0		9	4.4	4		1.3	**0.0285**
6C (*n*=36)	0	12	33.3	**<0.001**	1	0.5	11		3.5	**0.0272**
	1	1	2.8		1	0.5	0		0.0	0.2117
	2	0	0.0		0	0.0	0		0.0	N/A
	3	2	5.6		2	1.0	0		0.0	0.0770
	4	21	58.3		14	6.9	7		2.2	**0.0083**
6A (*n*=30)	0	23	76.7	**<0.001**	7	3.4	16		5.1	0.3600
	1	0	0.0		0	0.0	0		0.0	N/A
	2	1	3.3		1	0.5	0		0.0	0.2117
	3	1	3.3		1	0.5	0		0.0	0.2117
	4	5	16.7		5	2.5	0		0.0	**0.0050**
3 (*n*=23)	0	12	52.2	**<0.001**	0	0.0	12		3.8	**0.0047**
	1	2	8.7		0	0.0	2		0.6	0.2549
	2	0	0.0		0	0.0	0		0.0	N/A
	3	0	0.0		0	0.0	0		0.0	N/A
	4	9	39.1		8	3.9	1		0.3	**0.0022**
23F (*n*=21)	0	12	57.1	**<0.001**	1	0.5	11		3.5	**0.0272**
	1	2	9.5		0	0.0	2		0.6	0.2693
	2	1	4.8		0	0.0	1		0.3	0.4352
	3	0	0.0		0	0.0	0		0.0	N/A
	4	6	28.6		5	2.5	1		0.3	**0.0232**
19A (*n*=18)	0	11	61.1	**<0.001**	0	0.0	11		3.5	**0.0071**
	1	0	0.0		0	0.0	0		0.0	N/A
	2	0	0.0		0	0.0	0		0.0	N/A
	3	2	11.1		2	1.0	0		0.0	0.0770
	4	5	27.8		5	2.5	0		0.0	**0.0050**

Significant differences (*P*<0.05) are marked in bold.

a Chi-squared for Goodness of Fit.

b Test of proportion.

## Discussion

To the authors’ knowledge, this is the first study on nasopharyngeal carriage of *S. pneumoniae* in children in Serbia, and one of few worldwide, with the added value that it was performed in the unique situation of the COVID-19 pandemic. The pandemic led to the discontinuation of nasopharyngeal carriage monitoring in most countries. A significant increase in the prevalence of *S. pneumoniae* carriage was observed during the COVID-19 pandemic, with pneumococcal colonization being higher in fully vaccinated children and among those who attended day care centres. In addition, this study presented the first large dataset on *S. pneumoniae* carriage and serotype distribution according to the vaccination status of children and attendance of day care centres among children aged 24–60 months, 2–3 years since the introduction of PCV10 in the immunization programme in Serbia.

A substantial decline in IPD rates during the COVID-19 pandemic was observed in many countries, which was temporarily associated with lockdowns that included social distancing, masking and/or school closures (Brueggemann et al., 2021; Amin-Chowdhury et al.; 2021, McNeal et al., 2021; Danino et al., 2021; Perniciaro et al., 2022). In addition, the stringency of COVID-19 containment measures and changes in the movement of people appeared to coincide with the observed drop in cases of invasive disease, and a re-emergence of IPD has been observed in some countries since the relaxation of measures (Brueggemann et al., 2021; Perniciaro et al., 2022). Therefore, it is plausible to assume that such measures would also diminish person-to-person transmission of *S. pneumoniae* and, consequently, nasopharyngeal carriage of *S. pneumoniae* in children, as proposed by others (Cohen et al., 2021). However, data from the few studies that have addressed this issue to date suggest that this does not seem to be the case. In Belgium and Israel, the prevalence of pneumococcal carriage in children aged <3 years was either unchanged during the pandemic or was only reduced slightly, although the IPD rates decreased markedly in the same age group (Danino et al., 2021; Willen et al., 2022), Moreover, in Israel, colonization density and pneumococcal serotype distributions were similar to previous years (Danino et al., 2021). The present study did not find a decline in pneumococcal carriage rates among children aged 24–60 months during the COVID-19 pandemic, but instead found a significant increase in rates in 2020 that remained high to June 2021. Reasons for the discrepancy between the three studies could be different study settings (day care centres vs outpatient care facilities) and/or the composition of age groups of children (<3 years vs 24–60 months of age), although a significant increase was also detected in children aged 24–35 months in the present study. Also, SARS-CoV-2 co-infection could have impacted pneumococcal carriage rates in some children, as it has been shown that the nasopharyngeal carriage rate of *S. pneumoniae* in patients with COVID-19 was higher compared with that in non-infected children (Aykac et al., 2021; Howard, 2021). This was probably not the reason for the increased prevalence of *S. pneumoniae* carriage in the present study population, as only one colonized child had previous COVID-19 infection. Although the possibility that some of the tested children had asymptomatic and/or non-registered SARS-CoV-2 infection just before or at the moment of sampling cannot be excluded, it is unlikely that this was to such an extent to cause the observed increase in colonization rates among children in this study. On the other hand, attendance of day care centres may have affected high carriage rates in the latter stage of the pandemic in the study population. Indeed, the number of children attending day care centres during the pandemic in Novi Sad was in line with previous years except for the period of strict lockdown, which corresponded with the first period of sampling and the lowest *S. pneumoniae* carriage rates in this study. Later on, few restrictions were imposed, enabling intensive contacts between children and undisturbed transmission of *S. pneumoniae*, presumably leading to increased carriage rates (children aged <5 years were not obliged to wear face masks). Finally, as data on the prevalence of nasopharyngeal carriage of *S. pneumoniae* in children in Serbia before 2020 are not available, the possibility that the observed increase found in this study merely represented the return of the pneumoccocal carriage rate to the pre-pandemic level cannot be excluded. Nevertheless, given that a large number of samples were collected in February–March 2020, which coincided with the pre-pandemic/early pandemic period, it can be assumed that this time period is representative of the pre-PCV and/or pre-pandemic period (Table S1, see online supplementary material). Thus, it seems that this study found a true increase in the pneumoccocal carriage rate in children aged <5 years during the COVID-19 pandemic in Serbia. Whether this increase represents a local transient 'rebound' event after lifting of the COVID-19 containment measures, or a more general phenomenon that is occurring/will occur in other countries, and how will this affect the prevalence of IPD remains unclear, so further studies are needed. In line with this notion is the recent observation from Germany that, after an inital decline in the early months of the COVID-19 pandemic, the prevalence of IPD cases increased and exceeded the pre-pandemic level by June 2021 (Perniciaro et al., 2022).

Regarding the overall prevalence of *S. pneumoniae* colonization found in this study (31.7%), similar rates were obtained in other studies conducted before or immediately after the introduction of PCVs in children aged <2 years (31%), <5 years (35%) or <6 years (32%) during the pre-pandemic period (Moyo et al., 2012; Dayie et al., 2013; Emgård et al., 2019). The prevalence rate was higher in children aged 24–35 months than among those aged 36–60 months, which is consistent with many other studies (Darboe et al., 2010; Adetifa et al., 2012; Moyo et al., 2012; Dayie et al., 2013; Ueno et al., 2013; Ravi Kumar et al., 2014; Mills et al., 2015; Rutebemberwa et al., 2015; Menezes et al., 2016; Emgård et al., 2019).

In addition to age, other factors such as introduction of immunization practice with PCVs affect the rate of nasopharyngeal carriage in children, and play a key role in the distribution of certain serotypes in vaccinated children compared with unvaccinated children (Cui et al., 2017; World Health Organization, 2019). The trends and magnitude of change of vaccine-type carriage depend on the length of surveillance, and the impact of vaccination on *S. pneumoniae* carriage usually requires a long follow-up period (Andrade et al., 2014; Hammitt et al., 2014; Brandileone et al., 2016). The present study found that early *S. pneumoniae* carriage rates differed with regard to the age of participants and their vaccination status 2–3 years after the implementation of PCV10 in Serbia. The majority of children aged 36–60 months were unvaccinated, and the vaccine serotypes were more commonly identified in this age group compared with those aged 24–35 months. In contrast, the prevalence of non-vaccine serotypes of *S. pneumoniae* was higher in children aged 24–35 months than in children aged 36–60 months. These findings are not unexpected as it is known that PCVs may affect nasopharyngeal carriage of vaccine-related serotypes to some extent in vaccinated children, although this can vary between the vaccines, *S. pneumoniae* serotypes and children of different ages (McIntosh and Reinert, 2011; World Health Organization, 2012, 2019; Cui et al., 2017).

The predominant colonizing serotype in this study was 15B, as shown previously in children aged <5 years (Ceyhan et al., 2021). Serotypes 6B, 6C, 19F and 11A were also fairly common in both age groups. Contrary to children aged 36–60 months, serotypes 5, 7F and 9V (all present in PCV10) were not detected in children aged 24–35 months. Seven of the nine leading serotypes (6B, 19F, 11A, 6C, 23F, 3 and 19A) were significantly more prevalent in unvaccinated children aged 36–60 months compared with those aged 24–35 months, and all leading serotypes were significantly more prevalent among fully vaccinated children aged 24–35 months. Serotype 19A represented only 3.5% of all detected *S. pneumoniae* isolates, and its prevalence has not yet increased, unlike in many countries following PCV7 or PCV10 implementation where it emerged as one of the most prevalent serotypes in the post-vaccination era (McIntosh and Reinert, 2011; Cui et al., 2017; Lister et al., 2021).

This study had several limitations. First, the study was conducted in a single city with children from a highly urbanized area. Despite this, the major strength of the research lies in its long-term, prospective surveillance of both PCV uptake and *S. pneumoniae* carriage in an outpatient setting that included children treated at a single medical institution. Second, as mentioned previously, this research provided a snapshot of *S. pneumoniae* colonization and data were not available on pre-PCV carriage; as such, the results may underestimate the true impact of PCV10 on the prevalence of nasopharyngeal carriage and/or the distribution of vaccine serotypes. Third, this study included all children who presented to a medical doctor in the outpatient setting, regardless of whether or not they had any signs of upper respiratory tract infection. Although all participants who had a chronic and/or severe pathology were excluded, as well as those who had used antibiotics in the 7 days preceding sampling or during medical attendance, the possibility that the involvement of sick children led to a higher prevalence of *S. pneumoniae* carriage in this study cannot be excluded. However, it is reasonable to assume that the nasopharyngeal carriage rate of *S. pneumoniae* during the first days of acute respiratory infection was comparable to that in healthy children, as has been shown previously (Loughlin et al., 2014; Usonis et al., 2015; Daningrat et al., 2022). Fourth, due to the COVID-19 pandemic and the frequent interruption of sampling, it was not possible to draw firm conclusions about seasonal fluctuation and the prevalence of nasopharyngeal colonization of *S. pneumoniae*.

In conclusion, this study found that nasopharyngeal carriage of *S. pneumoniae* in children aged 24–60 months was high and increased during the COVID-19 pandemic, ruling out a major role of COVID-19 in the suppression of carriage and, probably, transmission. Implementation and constant improvement of pneumococcal surveillance is vital for the understanding of pneumococcal population dynamics, especially when facing a respiratory virus pandemic.

## Funding source

This research received funding from Pfizer (Grant No. 56659587) to the Research Fund of the Faculty of Medicine, University of Novi Sad. The funder had no role in the study design; in the data collection, analysis and interpretation of data; in the writing of the report, and in the decision to submit the article for publication.

## Ethical approval

The study protocol was reviewed and approved by the Ethics Committee of the Faculty of Medicine, University of Novi Sad (Reference No. 01-39/125/1). No authors of this study were involved in the treatment of patients included in the analysis, and all data were anonymized before being accessed by the authors.

## Declaration of Competing Interest

VP acts as the principal investigator for investigator-initiated sponsored studies related to the topic conducted on behalf of the Faculty of Medicine, University of Novi Sad, for which the Faculty obtained research grants from Pfizer and MSD. Outside the topic of work and in the last 36 months, VP, as an employee of the Faculty of Medicine, University of Novi Sads, obtained educational grants from Pfizer, GSK, MSD and Amicus; received paid fees for lectures from MSD, Pfizer and Sanofi Pasteur; served as a member of advisory boards for MSD, Sanofi Pasteur and Medison Pharma; and was a consultant at Expert Input Forums for MSD.

MM has received paid fees for lectures from Pfizer, MSD, Sanofi Pasteur, GSK and Amicus for presentations at symposia. Outside the topic of work and in the last 36 months, MM served as a member of advisory boards for Pfizer, Sanofi Pasteur, GSK and Medison Pharma; and was a consultant at Expert Input Forums for MSD.

Outside the topic of work and in the last 36 months, MR received paid fees for lectures from Sanofi Pasteur; and was a member of advisory boards for Sanofi Pasteur and Medison Pharma.

The other authors do not report any conflicts of interest.

## Appendix Supplementary materials

Image, application 1
