# Supplementary material for: Pneumococcal nasopharyngeal carriage in children under 5 years of age at an outpatient healthcare facility in Novi Sad, Serbia during the COVID-19 pandemic
Source: IJID Reg. 2022 Jul 7;4:88–96. doi: 10.1016/j.ijregi.2022.07.001 (PMC9294645; doi:10.1016/j.ijregi.2022.07.001)
Supplement: Supplementary file 1 [file mmc1.docx]

**Table S1**

Distribution of tested and laboratory-confirmed nasopharyngeal swabs for nasopharyngeal carriage during and within three sampling periods

| Year | Month | Tested | Positive | Prevalence (%) |
| --- | --- | --- | --- | --- |
| 2020 | February | 339 | 87 | 25.7 |
|  | March | 163 | 38 | 23.3 |
| Subtotal | | 502 | 125 | 24.9 |
| 2020 | September | 47 | 10 | 21.3 |
|  | October | 501 | 165 | 32.9 |
|  | November | 37 | 13 | 35.1 |
| Subtotal | | 585 | 188 | 32.1 |
| 2021 | April | 108 | 45 | 41.7 |
|  | May | 199 | 75 | 37.7 |
|  | June | 213 | 76 | 35.7 |
| Subtotal | | 520 | 196 | 37.7 |
| Subtotal (leading periods of sampling) | | 1607 | 509 | 31.7 |
| Total | | 1623 | 515 | 31.7 |
